# Ion-Gel-Assisted MoS_2_ Transfer Method for Low-Voltage, High-Performance MoS_2_/ITZO Heterojunction Phototransistor Application

**DOI:** 10.3390/mi17050574

**Published:** 2026-05-07

**Authors:** Soobin Lee, Jidong Jin, Zhenyuan Xiao, Wensi Cai, Zhigang Zang, Hyun Seok Lee, Jaekyun Kim

**Affiliations:** 1 Department of Photonics and Nanoelectronics, Hanyang University, Ansan 15588, Republic of Korea; 2 Key Laboratory of Optoelectronic Technology & Systems (Ministry of Education), Chongqing University, Chongqing 400044, China; 3 Department of Physics, Chungbuk National University, Cheongju 28644, Republic of Korea

**Keywords:** phototransistor, MoS_2_, large-area transfer, ion gel, low voltage

## Abstract

Molybdenum disulfide (MoS_2_) is a compelling candidate for visible-light detection due to its strong optical absorption and tunable bandgap, yet the development of high-performance MoS_2_ photodetectors remains limited by challenges in scalable integration, low-voltage operation, and efficient photoresponse. Here, we report an ion-gel-assisted transfer strategy that enables the fabrication of large-area MoS_2_/ion gel films that are suitable for low-power phototransistor applications. The transferred MoS_2_/ion gel stack is laminated onto an indium-tin-zinc-oxide (ITZO) layer on a glass substrate to fabricate a MoS_2_/ITZO heterojunction phototransistor, with the ion gel serving as an ultrathin, high-capacitance gate dielectric. The resulting phototransistor exhibits a field-effect mobility of 4.12 cm^2^/Vs, an on/off current ratio of 4.9 × 10^5^, and a subthreshold swing of 0.17 V/dec. Under 635, 520, and 405 nm illumination with a power density of 4.5 mW/cm^2^, it achieves responsivities of 0.58, 1.82, and 5.56 A W^−1^ and detectivities of 5.90 × 10^9^, 1.86 × 10^10^, and 5.68 × 10^10^ Jones, respectively. These findings demonstrate that the ion-gel-assisted transfer process offers a robust route to high-performance, low-voltage photodetection and provides a promising platform for next-generation optoelectronic technologies.

## 1. Introduction

Two-dimensional (2D) materials have been extensively investigated for a wide range of applications, including optoelectronics, photonics, sensing, and energy storage, due to their excellent optical and electrical properties [1,2,3,4,5]. Among these materials, molybdenum disulfide (MoS_2_) has received significant attention in the field of optoelectronics, benefiting from its tunable bandgap, high carrier mobility, and excellent absorption capabilities [6]. By controlling the number of layers, the bandgap of MoS_2_ can be adjusted, transitioning from 1.29 eV (bulk) to 1.80 eV (monolayer), resulting in a shift from an indirect to a direct bandgap due to quantum confinement effects [7]. MoS_2_-based phototransistors have demonstrated very high photoresponsivity and detectivity [8,9,10,11,12]. Notably, MoS_2_ can be effectively integrated with a range of oxide semiconductors to form high-performance heterojunction phototransistors [12,13]. Recently, Jin et al. reported an IGZO/MoS_2_ heterojunction phototransistor that outperforms standalone MoS_2_ devices because the IGZO layer provides additional photocarriers primarily through the photoionization of oxygen-vacancy-related defect states. These supplementary carriers, combined with the enhanced electrostatic modulation enabled by the heterojunction, strengthen the photogating effect and ultimately lead to improved responsivity and detectivity.

Phototransistors based on mechanically exfoliated MoS_2_ flakes have demonstrated remarkable photoresponsivity [14]. However, such flakes are not suitable for large-area electronic applications. Consequently, substantial research efforts have focused on developing alternative large-area deposition methods for MoS_2_ thin films, including sputtering [10,15,16], atomic layer deposition [17], and chemical vapor deposition (CVD) [18,19,20]. Nevertheless, the as-grown MoS_2_ films produced by these methods often require transfer to a target substrate for subsequent device fabrication [21].

The transfer of MoS_2_ films has been extensively investigated using a polymer supporting layer, such as polymethylmethacrylate (PMMA) and polydimethylsiloxane (PDMS) [21,22]. PMMA-assisted transfer is the most widely used and established technique, enabling precise control of the transfer process [21]. However, it commonly requires harsh chemical etchants to remove the growth substrate, which can introduce cracks, wrinkles, and polymer residues in the MoS_2_ film [21,22,23]. In contrast, PDMS-assisted transfer offers a simpler, etchant-free alternative [21,24]. In this approach, a PDMS layer is applied to the as-grown MoS_2_ and mechanically peeled off due to surface-energy differences. Successful transfer requires that the MoS_2_–substrate adhesion exceed that of the PDMS–MoS_2_ interface, where higher adhesion corresponds to higher surface energy [25]. However, mechanical detachment of PDMS after transfer can result in incomplete film release [21,26]. To mitigate this limitation, strategies such as surface-energy modification of PDMS [27] or water-assisted lift-off processes [24] have been introduced.

These limitations highlight the importance of engineering interfacial adhesion during transfer. Recent studies on adhesion-assisted transfer printing for nanoparticle monolayers [28] and liquid metal particle films [29] have demonstrated that controlled adhesion can enable reliable pickup and release. However, extending such strategies to continuous 2D materials like MoS_2_ poses additional challenges. Unlike discrete nanoparticles, MoS_2_ forms an atomically thin yet laterally continuous film, making it highly susceptible to wrinkling, cracking, and contamination during transfer. Therefore, precise control of adhesion is essential to maintain film integrity.

In this study, we employ an ion-gel-assisted transfer method for MoS_2_ that is enhanced through surface-energy modification. Specifically, an ion gel intermediate layer is introduced between PDMS and MoS_2_ to increase the interfacial adhesion to MoS_2_. This surface-energy-assisted approach effectively overcomes the intrinsically weak adhesion at the PDMS–MoS_2_ interface, enabling more reliable and complete film transfer. Initially, ion gel solutions are drop-casted onto the as-grown MoS_2_ film on SiO_2_/Si and then attached to the PDMS stamp. Remarkably, the ion gel film exhibits strong adhesion to MoS_2_ after curing with UV exposure (354 nm, 150 mW/cm^2^). Following curing, the PDMS stamp can be detached, resulting in the MoS_2_/ion gel film being mechanically peeled off without any wrinkles, cracks, or polymer residues. By employing this ion-gel-assisted transfer method, we achieve perfect transfer of MoS_2_/ion gel films onto the target substrate to fabricate ITZO/MoS_2_ heterojunction phototransistors. Importantly, the ion gel fulfills a dual role, acting as the transfer medium for MoS_2_ and the gate dielectric for the phototransistor. The integration of ITZO and MoS_2_ significantly enhances photosensitivity, thereby establishing a promising foundation for the development of high-performance phototransistors.

## 2. Materials and Methods

### 2.1. MoS_2_ Synthesis

The solution consisted of two Mo-precursors: ammonium molybdate tetrahydrate (AMT, Sigma-Aldrich, St. Louis, MO, USA), and sodium molybdate dihydrate (SMD, Sigma Aldrich), along with a promoter, NaOH (#306576, Sigma Aldrich), and a surfactant, iodixanol (Opti, Sigma Aldrich). These mixtures were dissolved in deionized (DI) water, with a mixing ratio of NaOH: SMD = 3:0.25 and AMT: Opti = 1:0.25. The liquid precursor solution was then dropped onto the SiO_2_/Si substrate, followed by a spin-coating process at 1000 rpm for 30 s. For synthesis, the sulfur-precursor source (S powder, Sigma-Aldrich) was placed in a ceramic boat in zone 1, while the liquid-solution-coated substrate was placed in zone 2 of the CVD chamber. Zone 1 and zone 2 were heated to 320 and 800 °C, respectively. Subsequently, with N_2_ carrier gas flowing at a rate of 200 sccm, the sublimated S from zone 1 reacted with the spin-coated film in zone 2, resulting in the successful synthesis of MoS_2_ film.

### 2.2. Ion Gel Synthesis

The UV-curable precursor was formulated by combining poly(ethylene glycol) diacrylate (PEGDA) and the photoinitiator 2-hydroxy-2-methylpropiophenone (HOMPP) at a weight ratio of 2:1, followed by stirring for 12 h. To introduce ionic conductivity, 1-ethyl-3-methylimidazolium bis(trifluoromethylsulfonyl)imide (EMIM-TFSI) was incorporated into the UV–polymer solution at 50 wt%. The resulting ion gel mixture was stirred until a uniform and fully homogeneous solution was obtained.

### 2.3. Ion-Gel-Assisted Transfer Process

**Figure 1:**
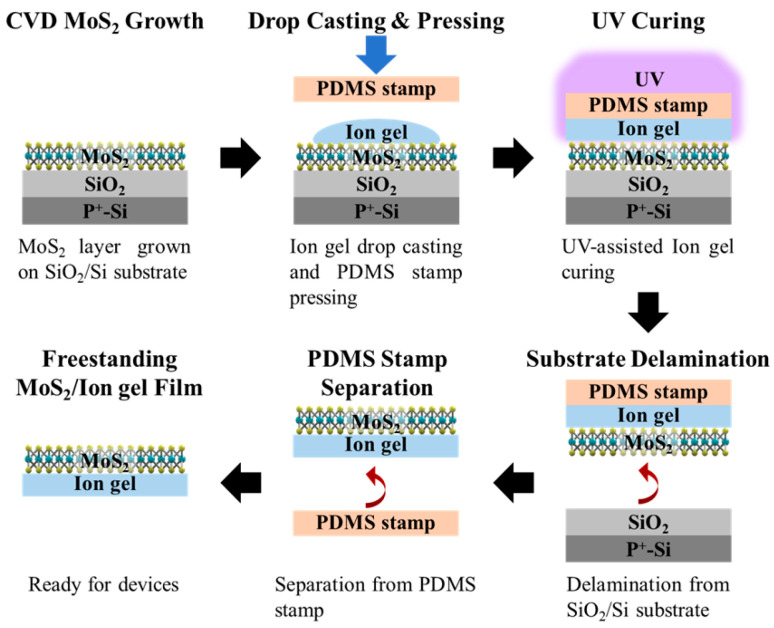
MoS_2_/ion gel freestanding film fabrication using the ion-gel-assisted transfer process.

First, the MoS_2_ layer was grown on a SiO_2_/Si substrate via CVD. The ion gel solution was then drop-cast onto the MoS_2_ and pressed with a PDMS stamp, forming a 60 μm ion gel film between MoS_2_ and PDMS. The PDMS stamp was prepared according to a previously reported method [30]. Subsequently, UV light exposure (354 nm, 150 mW/cm^2^) was employed to cure the ion gel film. The transfer is governed by interfacial adhesion [31]: owing to its liquid-rich and compliant nature, the ion gel forms intimate contact with MoS_2_, resulting in stronger adhesion at the MoS_2_/ion gel interface than at the MoS_2_/SiO_2_ interface. Consequently, the MoS_2_/ion gel/PDMS stack can be mechanically delaminated from the SiO_2_/Si substrate. Finally, the PDMS stamp can be readily peeled off due to its intrinsically low surface energy and weak interfacial interaction with the ion gel [31,32], yielding a freestanding MoS_2_/ion gel film for subsequent phototransistor fabrication. The detailed steps of this ion-gel-assisted transfer process are illustrated in Figure 1.

### 2.4. Fabrication of MoS_2_/ITZO Phototransistor

First, a 10 nm ITZO film was deposited on glass substrates by RF sputtering, using an ITZO target (In_2_O_3_: SnO_2_: ZnO = 4:1:4 mol%) at room temperature. The deposition was carried out with RF power of 25 W in an Ar gas ambient. The as-deposited ITZO film was subsequently annealed in air at 250 °C for 1 h. Next, 50 nm Al source/drain (S/D) electrodes were formed by thermal evaporation. The width and length of the ITZO channel were 1000 and 50 μm, respectively. Then, the MoS_2_/ion gel film, obtained using the ion-gel-assisted transfer method, was laminated onto the ITZO layer with pre-patterned Al S/D electrodes. Finally, a silver (Ag) paste was applied on top of the ion gel film to form the top gate electrode of the ITZO/MoS_2_ phototransistor.

### 2.5. Device and Film Characterization

The transfer and output characteristics of the device were measured using a semiconductor parameter analyzer (4200 SCS, Keithley, Solon, OH, USA). Raman and photoluminescence (PL) spectra were performed confocal Raman/PL system with a 532 nm laser (Xperam S500, Nanobase, Seoul, Republic of Korea). A UV–Vis spectrophotometer was used to obtain the optical bandgap of ITZO film (Mega-V600, Scinco, Seoul, Republic of Korea). The specific capacitance of the ion gel film was measured using an LCR meter (E4980A, Keithley). All measurements were carried out in ambient air at room temperature.

## 3. Results and Discussion

**Figure 2:**
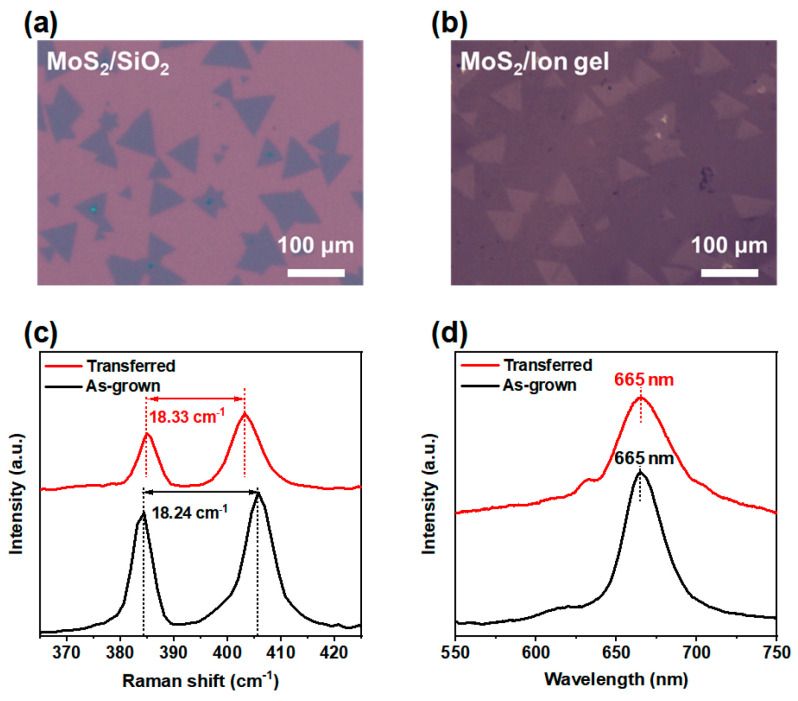
The optical microscope images corresponding to (**a**) as-grown MoS_2_, (**b**) transferred MoS_2_/ion gel freestanding film. (**c**) Raman and (**d**) PL spectra of MoS_2_ film before and after the transfer.

The optical microscope images of as-grown and transferred MoS_2_ flakes are depicted in Figure 2a and Figure 2b, respectively. The transferred MoS_2_ film presents a smooth surface without any wrinkles or cracks. Figure 2c displays the Raman spectroscopy of both as-grown MoS_2_ film and MoS_2_/ion gel film obtained via the transfer method. The frequency difference between the two Raman modes (E^1^_2g_ and A_1g_) corresponding to as-grown MoS_2_ film is 18.24 cm^−1^, indicating a monolayer film [33]. The transferred MoS_2_ film shows a frequency difference of 18.33 cm^−1^, along with shifts of 0.52 cm^−1^ for the E^1^_2g_ peak and 0.43 cm^−1^ for the A_1g_ peak, relative to the original peak value of the as-grown MoS_2_ film. These slight changes in the peak positions may be attributed to variations in substrate-induced strain or charge transfer and electronic interactions between the MoS_2_ film and different substrates [34]. Both the as-grown and transferred MoS_2_ exhibit a dominant peak at approximately 665 nm (Figure 2d), indicative of a 1.86 eV band gap.

**Figure 3:**
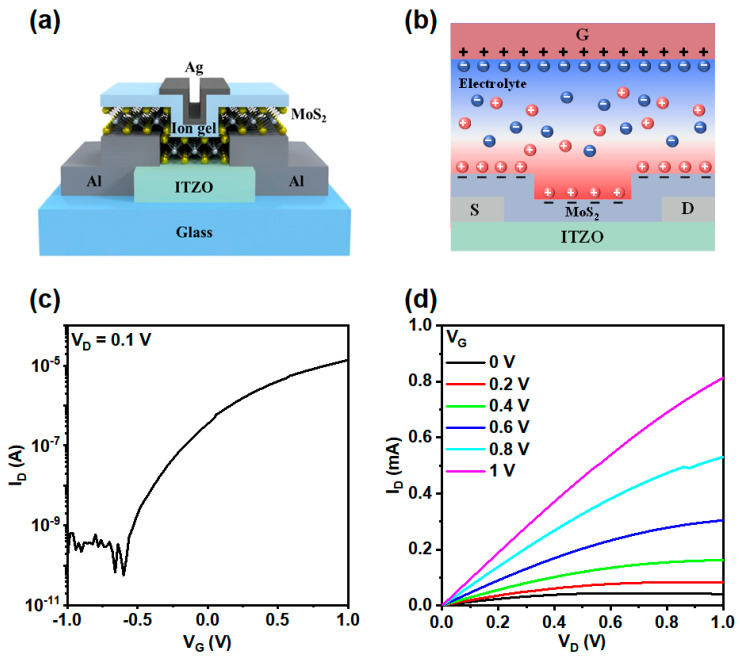
(**a**) Schematic illustration of the ion-gel-gated MoS_2_/ITZO TFT. (**b**) EDL-gating mechanism under a positive gate voltage. (**c**) Transfer and (**d**) output characteristics of the ITZO/MoS_2_ TFT.

Figure 3a shows a schematic illustration of a phototransistor featuring an ion gel-gated ITZO/MoS_2_ heterojunction. The detailed fabrication process is described in the experimental section. Here, the ion gel serves as both the transfer medium for MoS_2_ and the gate dielectric for the phototransistor. The ion gel dielectric enables low-voltage operation by forming an electric double layer (EDL), which is capable of accumulating a significant amount of charge, resulting in a high capacitance [35,36]. Figure 3b schematically illustrates the EDL-gating mechanism under a positive gate voltage (*V_G_*). When a positive V_G_ is applied, cations in the ion gel migrate toward and accumulate at the MoS_2_/ion gel interface, while anions move toward the gate electrode. Applying a negative V_G_ reverses the direction of ion migration, leading to opposite ionic accumulation at the respective interfaces. The frequency-dependent specific capacitance of the ion gel is measured using a metal/ion gel/metal structure, as shown in Figure S1. Notably, the ion gel exhibits a maximum specific capacitance of 3.41 μF/cm^2^ at 50 Hz. As the frequency increases, the capacitance decreases, which is attributed to the ion mobilities restrict the polarization response time at higher frequencies [30]. The single-sweep transfer and output characteristics of the phototransistors are shown in Figure 3c and Figure 3d, respectively. A dual-sweep transfer curve, highlighting the hysteresis behavior, is provided in Figure S2. As expected, the phototransistor enables ultra-low voltage operation at 1 V, due to the high specific capacitance of the ion gel dielectric film. The field-effect mobility (*µ_FE_*) and subthreshold swing (*SS*) of the ITZO/MoS_2_ device were obtained from the transfer curve using the following equations [37]:

(1) $$\mu_{FE}=\frac{g_{m}L}{C_{ox}WV_{D}}$$

(2) $$\mathrm{SS}={\left(\frac{d\log{(I}_{D})}{dV_{G}}{|}_{\max}\right)}^{-1}$$

where *L* is the channel length, *W* is the channel width, *C_ox_* is the capacitance per unit area, *V_D_* is the drain voltage, *g_m_* is the transconductance, and *I_D_* is the drain current. The ITZO/MoS_2_ device shows a *µ_FE_* of 4.12 cm^2^/Vs, an *SS* of 0.17 V/dec, an on/off current ratio of 4.9 × 10^5^, and a low turn-on voltage (*V_on_*) of −0.59 V.

**Figure 4:**
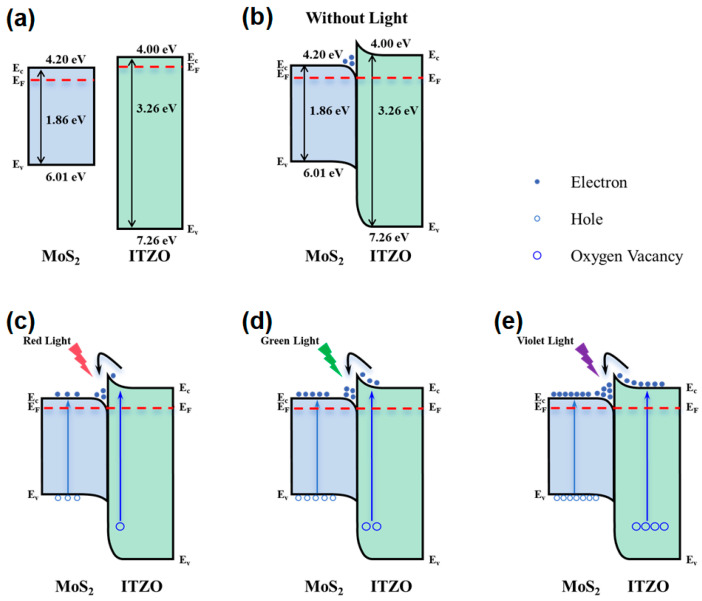
(**a**) Energy band diagram of bilayer ITZO/MoS_2_ channel before contact, (**b**) after contact without light illumination, (**c**) after contact with red light, (**d**) after contact with green light, and (**e**) after contact with violet light.

We conducted an assessment of optical transmittance using an UV–Vis spectrometer to derive the bandgap of ITZO film for the phototransistor. The bandgap of ITZO can be related to absorption coefficient by the following equation [38]:

(3) $$\alpha hv={\left(hv-E_{g}\right)}^{\frac{1}{2}}$$

where α is the absorption coefficient, *h* is the Planck constant, *v* is the incident photon frequency, and *E_g_* is the bandgap. Based on the plots of (*ahv*)^2^ against photon energy *hv*, the band gap is determined by extrapolating from the straight-line part of the plot (see Figure S3). This extrapolation yields an ITZO band gap of 3.26 eV. The electron affinity of MoS_2_ is approximately 4.2 eV [39], while that of ITZO is estimated to be around 4.0 eV based on a previous report [40]. Using these values, the ITZO/MoS_2_ energy band diagrams before and after heterojunction formation were constructed, as shown in Figure 4. As illustrated in Figure 4b, a large conduction-band offset at the ITZO/MoS_2_ interface creates a potential well that confines electrons at the heterojunction without light illumination. Figure 4c–e illustrate the wavelength-dependent photo response of the devices under red (635 nm, 1.95 eV), green (520 nm, 2.38 eV), and violet (406 nm, 3.05 eV) illumination. Under illumination, these electrons from both MoS_2_ and ITZO accumulate in the heterojunction potential well. Photons with energies above the MoS_2_ bandgap generate electron–hole pairs in the MoS_2_ channel layer, while oxygen vacancies (V_O_) in the ITZO layer can be photoionized, releasing electrons to the conduction band and converting to Vo^+^ or Vo^2+^ [41]. Under red-light illumination, the photocurrent originates primarily from electron–hole pairs generated in the MoS_2_ layer. The ITZO layer contributes minimally because the photon energy (1.95 eV) is insufficient to significantly ionize V_O_. Under green-light illumination, which provides a higher photon energy (2.38 eV), the photocurrent still mainly arises from MoS_2_, but the ITZO side contributes slightly more due to the increased photoionization of V_O_. In contrast, violet light delivers the highest photon energy (3.05 eV), enabling photo-generated electrons from both MoS_2_ and ITZO to accumulate within the heterojunction potential well.

**Figure 5:**
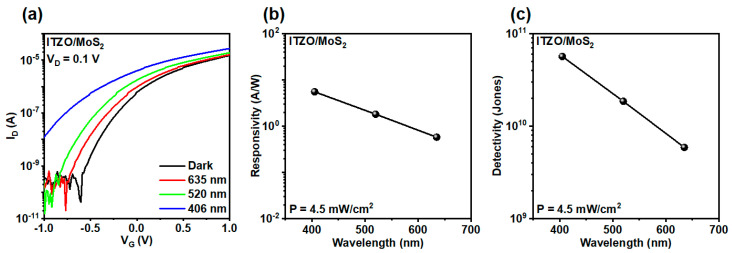
(**a**) Transfer curves of the ITZO/MoS_2_ transistor under different light illuminations with a fixed power density of 4.5 mW/cm^2^. (**b**) Responsivity and (**c**) detectivity as a function of different light wavelength.

The transfer characteristics of the ITZO/MoS_2_ transistor measured under dark conditions and under illumination with red, green, and violet light (power density: 4.5 mW/cm^2^) are shown in Figure 5a. Owing to the pronounced hysteresis of the ion-gel-gated phototransistor (Figure S2), arising from ionic polarization [42], the photoresponse was evaluated under fixed-bias conditions based on single-sweep measurements to reduce sweep-direction-dependent variations. Relative to the dark state, illumination induces a substantial increase in the drain current, with a distinct wavelength-dependent modulation that indicates effective photogating and carrier generation within the heterostructure. The responsivity (*R*) and detectivity (D*) of the ITZO/MoS_2_ transistor were determined using the following equations [13]:

(4) $$R=\frac{I_{ph}}{PA}$$

(5) $$D^{*}=\frac{A^{1/2}R}{{\left(2eI_{dark}\right)}^{1/2}}$$

where *I_ph_* is photocurrent, which is defined as the difference between the drain current under illumination and the drain in the dark, *P* is the incident light power density, *A* is the device active area, and *e* is the elementary electron charge. The phototransistor exhibits *R* of 0.58, 1.82, and 5.56 A W^−1^ at illumination wavelengths of 635, 520, and 405 nm, respectively, as shown in Figure 5b. Despite lower photoresponsivity compared to high-bias phototransistors [14], our device operates at an ultralow voltage of 1 V, enabled by ion gel gating. This low-voltage operation significantly reduces power consumption and highlights the efficiency of EDL gating in modulating the channel at low bias.

The responsivity increases with decreasing wavelength, consistent with the higher photon energies that generate more electron–hole pairs in the MoS_2_ channel and more effectively induce the photoionization of oxygen vacancies in the ITZO layer. Consistent with this trend, the D* reaches 5.90 × 10^9^, 1.86 × 10^10^, and 5.68 × 10^10^ Jones at the same wavelengths (Figure 5c). These values highlight the device’s broadband response and demonstrate that the highest detection performance occurs under violet illumination, where both intrinsic MoS_2_ absorption and defect-assisted photogeneration in the ITZO are maximized.

## 4. Conclusions

In summary, we demonstrated an ion-gel-assisted transfer strategy for fabricating large-area MoS_2_/ion gel films optimized for low-power phototransistor applications. By laminating the transferred MoS_2_/ion gel stack onto an ITZO layer, we fabricated a high-quality MoS_2_/ITZO heterojunction phototransistor incorporating an ultrathin, high-capacitance ion-gel-gated dielectric. The resulting devices exhibited excellent electrical and optoelectronic characteristics, including a *µ_FE_* of 4.12 cm^2^/Vs, an on/off current ratio of 4.9 × 10^5^, and an *SS* of 0.17 V/dec. Under illumination at 635, 520, and 405 nm with a power density of 4.5 mW/cm^2^, the phototransistor achieved responsivities of 0.58, 1.82, and 5.56 A W^−1^ and detectivities of 5.90 × 10^9^, 1.86 × 10^10^, and 5.68 × 10^10^ Jones, respectively. These results validate the effectiveness of the ion-gel-assisted process for enabling high-performance, low-voltage photodetection, and highlight its potential as a scalable platform for next-generation optoelectronic technologies.

## Data Availability

The original contributions presented in this study are included in the article. Further inquiries can be directed to the corresponding authors.

## Supplementary Materials

The following supporting information can be downloaded at https://www.mdpi.com/article/10.3390/mi17050574/s1: Figure S1: Specific capacitance characteristics of Al-ion gel-Al device. Figure S2: Dual-sweep transfer characteristic of the ITZO/MoS_2_ TFT. Figure S3: Optical bandgap spectra of the ITZO film.

## References

[1] Shim J., Park H.-Y., Kang D.-H., Kim J.-O., Jo S.-H., Park Y., Park J.-H. (2017). Electronic and Optoelectronic Devices based on Two-Dimensional Materials: From Fabrication to Application. Adv. Electron. Mater..

[2] Choi W., Choudhary N., Han G.H., Park J., Akinwande D., Lee Y.H. (2017). Recent development of two-dimensional transition metal dichalcogenides and their applications. Mater. Today.

[3] Mueller T., Malic E. (2018). Exciton physics and device application of two-dimensional transition metal dichalcogenide semiconductors. npj 2D Mater. Appl..

[4] Rohaizad N., Mayorga-Martinez C.C., Fojtů M., Latiff N.M., Pumera M. (2021). Two-dimensional materials in biomedical, biosensing and sensing applications. Chem. Soc. Rev..

[5] Sangeetha P., Ayyanar N., Prabhakar G., Rajaram S. (2025). Study Review of Optical Biosensors Based on 2D Materials. Plasmonics.

[6] Nalwa H.S. (2020). A review of molybdenum disulfide (MoS_2_) based photodetectors: From ultra-broadband, self-powered to flexible devices. RSC Adv..

[7] Mak K.F., Lee C., Hone J., Shan J., Heinz T.F. (2010). Atomically thin MoS_2_: A new direct-gap semiconductor. Phys. Rev. Lett..

[8] Kufer D., Konstantatos G. (2015). Highly Sensitive, Encapsulated MoS_2_ Photodetector with Gate Controllable Gain and Speed. Nano Lett..

[9] Lee Y.T., Kang J.-H., Kwak K., Ahn J., Choi H.T., Ju B.-K., Shokouh S.H., Im S., Park M.-C., Hwang D.K. (2018). High-Performance 2D MoS_2_ Phototransistor for Photo Logic Gate and Image Sensor. ACS Photonics.

[10] Park H., Liu N., Kim B.H., Kwon S.H., Baek S., Kim S., Lee H.-K., Yoon Y.J., Kim S. (2020). Exceptionally Uniform and Scalable Multilayer MoS_2_ Phototransistor Array Based on Large-Scale MoS_2_ Grown by RF Sputtering, Electron Beam Irradiation, and Sulfurization. ACS Appl. Mater. Interfaces.

[11] Choi W., Cho M.Y., Konar A., Lee J.H., Cha G.B., Hong S.C., Kim S., Kim J., Jena D., Joo J. (2012). High-detectivity multilayer MoS_2_ phototransistors with spectral response from ultraviolet to infrared. Adv. Mater..

[12] Yang J., Kwak H., Lee Y., Kang Y.-S., Cho M.-H., Cho J.H., Kim Y.-H., Jeong S.-J., Park S., Lee H.-J. (2016). MoS_2_–InGaZnO Heterojunction Phototransistors with Broad Spectral Responsivity. ACS Appl. Mater. Interfaces.

[13] Jin J., Kang K., Xiao Z., Zhang J., Kim T.Y., Lee H.S., Kim J. (2025). Improved Process Stability and Light Detection in Phototransistors via Inverted MoS_2_/a-IGZO Heterojunction Integration. Phys. Status Solidi A.

[14] Lopez-Sanchez O., Lembke D., Kayci M., Radenovic A., Kis A. (2013). Ultrasensitive photodetectors based on monolayer MoS_2_. Nat. Nanotechnol..

[15] Tao J., Chai J., Lu X., Wong L.M., Wong T.I., Pan J., Xiong Q., Chi D., Wang S. (2015). Growth of wafer-scale MoS_2_ monolayer by magnetron sputtering. Nanoscale.

[16] Kim R.H., Leem J., Muratore C., Nam S., Rao R., Jawaid A., Durstock M., McConney M., Drummy L., Rai R. (2019). Photonic crystallization of two-dimensional MoS_2_ for stretchable photodetectors. Nanoscale.

[17] Keller B.D., Bertuch A., Provine J., Sundaram G., Ferralis N., Grossman J.C. (2017). Process Control of Atomic Layer Deposition Molybdenum Oxide Nucleation and Sulfidation to Large-Area MoS_2_ Monolayers. Chem. Mater..

[18] Bilgin I., Liu F., Vargas A., Winchester A., Man M.K.L., Upmanyu M., Dani K.M., Gupta G., Talapatra S., Mohite A.D. (2015). Chemical Vapor Deposition Synthesized Atomically Thin Molybdenum Disulfide with Optoelectronic-Grade Crystalline Quality. ACS Nano.

[19] Zhu D., Shu H., Jiang F., Lv D., Asokan V., Omar O., Yuan J., Zhang Z., Jin C. (2017). Capture the growth kinetics of CVD growth of two-dimensional MoS_2_. npj 2D Mater. Appl..

[20] He T., Li Y., Zhou Z., Zeng C., Qiao L., Lan C., Yin Y., Li C., Liu Y. (2019). Synthesis of large-area uniform MoS_2_ films by substrate-moving atmospheric pressure chemical vapor deposition: From monolayer to multilayer. 2D Mater..

[21] Watson A.J., Lu W., Guimarães M.H., Stöhr M. (2021). Transfer of large-scale two-dimensional semiconductors: Challenges and developments. 2D Mater..

[22] Ma D., Shi J., Ji Q., Chen K., Yin J., Lin Y., Zhang Y., Liu M., Feng Q., Song X. (2015). A universal etching-free transfer of MoS_2_ films for applications in photodetectors. Nano Res..

[23] Sharma M., Singh A., Aggarwal P., Singh R. (2022). Large-Area Transfer of 2D TMDCs Assisted by a Water-Soluble Layer for Potential Device Applications. ACS Omega.

[24] Thakur M., Macha M., Chernev A., Graf M., Lihter M., Deen J., Tripathi M., Kis A., Radenovic A. (2020). Wafer-Scale Fabrication of Nanopore Devices for Single-Molecule DNA Biosensing using MoS_2_. Small Methods.

[25] Johnson K.L., Kendall K., Roberts A.D. (1971). Surface energy and the contact of elastic solids. Proc. R. Soc. A Math. Phys. Sci..

[26] Gurarslan A., Yu Y., Su L., Yu Y., Suarez F., Yao S., Zhu Y., Ozturk M., Zhang Y., Cao L. (2014). Surface-Energy-Assisted Perfect Transfer of Centimeter-Scale Monolayer and Few-Layer MoS_2_ Films onto Arbitrary Substrates. ACS Nano.

[27] Kang M.-A., Kim S.J., Song W., Chang S.-j., Park C.-Y., Myung S., Lim J., Lee S.S., An K.-S. (2017). Fabrication of flexible optoelectronic devices based on MoS_2_/graphene hybrid patterns by a soft lithographic patterning method. Carbon.

[28] Li X., Chen L., Ma Y., Weng D., Li Z., Song L., Zhang X., Yu G., Wang J. (2022). Ultrafast Fabrication of Large-Area Colloidal Crystal Micropatterns via Self-Assembly and Transfer Printing. Adv. Funct. Mater..

[29] Li X., Rytkin E., Zhao Q., Bhat P., Pfenniger A., Yin L., Huang X., Yang L., Yang B., Burrell A. (2025). High-resolution liquid metal–based stretchable electronics enabled by colloidal self-assembly and microtransfer printing. Sci. Adv..

[30] Lee D., Song Y.H., Choi U.H., Kim J. (2019). Highly Flexible and Stable Solid-State Supercapacitors Based on a Homogeneous Thin Ion Gel Polymer Electrolyte Using a Poly(dimethylsiloxane) Stamp. ACS Appl. Mater. Interfaces.

[31] Lee K.H., Zhang S., Gu Y., Lodge T.P., Frisbie C.D. (2013). Transfer Printing of Thermoreversible Ion Gels for Flexible Electronics. ACS Appl. Mater. Interfaces.

[32] Vudayagiri S., Junker M.D., Skov A.L. (2013). Factors affecting the surface and release properties of thin polydimethylsiloxane films. Polym. J..

[33] Liu Y., Nan H., Wu X., Pan W., Wang W., Bai J., Zhao W., Sun L., Wang X., Ni Z. (2013). Layer-by-Layer Thinning of MoS_2_ by Plasma. ACS Nano.

[34] Buscema M., Steele G.A., van der Zant H.S.J., Castellanos-Gomez A. (2014). The effect of the substrate on the Raman and photoluminescence emission of single-layer MoS_2_. Nano Res..

[35] Lee J., Kaake L.G., Cho J.H., Zhu X.Y., Lodge T.P., Frisbie C.D. (2009). Ion Gel-Gated Polymer Thin-Film Transistors: Operating Mechanism and Characterization of Gate Dielectric Capacitance, Switching Speed, and Stability. J. Phys. Chem. C.

[36] Xu K., Liang J., Woeppel A., Bostian M.E., Ding H., Chao Z., McKone J.R., Beckman E.J., Fullerton-Shirey S.K. (2019). Electric Double-Layer Gating of Two-Dimensional Field-Effect Transistors Using a Single-Ion Conductor. ACS Appl. Mater. Interfaces.

[37] Zhong W., Kang L., Deng S., Lu L., Yao R., Lan L., Kwok H.S., Chen R. (2021). Effect of Sc_2_O_3_ Passivation Layer on the Electrical Characteristics and Stability of InSnZnO Thin-Film Transistors. IEEE Trans. Electron Devices.

[38] Yang H., Yang W., Su J., Zhang X. (2022). Enhancement-mode thin film transistor using amorphous phosphorus-doped Indium–Zinc–Tin-Oxide channel layer. Mater. Sci. Semicond. Process..

[39] Furchi M.M., Pospischil A., Libisch F., Burgdörfer J., Mueller T. (2014). Photovoltaic Effect in an Electrically Tunable van der Waals Heterojunction. Nano Lett..

[40] Xiao Z., Le Q.T., Lv S., Song A., Zhang J., Jin J., Kim J. (2025). Enhanced Carrier Transport and Stability in Thin-Film Transistors Based on ITZO/IGZO Heterojunction Channel and Al_2_O_3_ Passivation. ACS Appl. Electron. Mater..

[41] Xiao Z., Jin J., Lee J., Choi G., Lin X., Zhang J., Kim J. (2024). Improved Performance and Bias Stability of Indium-Tin-Zinc-Oxide Thin-Film Transistors Enabled by an Oxygen-Compensated Capping Layer. Phys. Status Solidi A.

[42] Wang D., Zhao S., Yin R., Li L., Lou Z., Shen G. (2021). Recent advanced applications of ion-gel in ionic-gated transistor. npj Flex. Electron..

