# Supplementary material for: Ion-Gel-Assisted MoS2 Transfer Method for Low-Voltage, High-Performance MoS2/ITZO Heterojunction Phototransistor Application
Source: Micromachines (Basel). 2026 May 7;17(5):574. doi: 10.3390/mi17050574 (PMC13208767; doi:10.3390/mi17050574)
Supplement: Supplementary file 1 [file micromachines-17-00574-s001.zip › micromachines-4231632-supplementary.pdf]

Supporting Information

Ion-Gel-Assisted MoS_2 Transfer Method for Low-Voltage, High-Performance MoS_2/ITZO Heterojunction Phototransistor

Soobin Lee [1,†], Jidong Jin [1,†], Zhenyuan Xiao [1], Wensi Cai [2], Zhigang Zang [2], Hyun Seok Lee [3,*], and Jaekyun Kim [1,*]

[1]Deparment of Photonics and Nanoelectronics, Hanyang University, Ansan 15588, Republic of Korea

[2]Key Laboratory of Optoelectronic Technology & Systems (Ministry of Education), Chongqing University, Chongqing 400044, China

[3]Department of Physics, Chungbuk National University, Cheongju 28644, Republic of Korea

[†]Equal contribution

*Correspondence: hsl@chungbuk.ac.kr (H.S.L.) jaekyunkim@hanyang.ac.kr (J.K.);

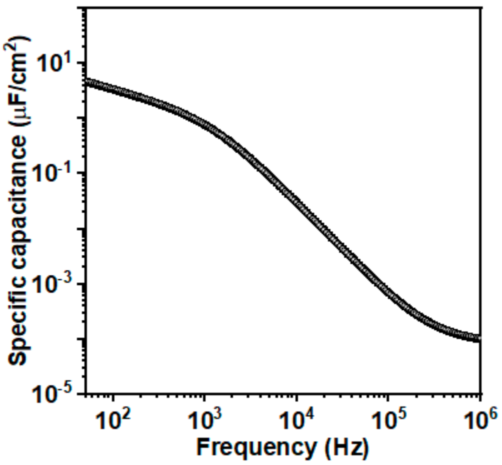

Figure S1. Specific capacitance characteristics of the Al/ion-gel/Al device.

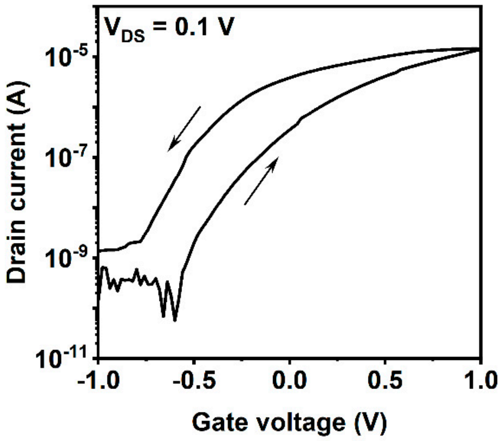

Figure S3**.** Dual-sweep transfer characteristic of the ITZO/MoS_2 TFT, demonstrating hysteresis during forward and reverse gate-voltage sweeps.

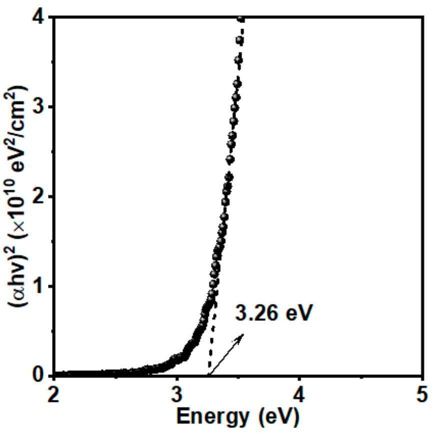

Figure S3**.** Optical bandgap spectra of the ITZO film.
